# Revising Terminology and Phenotype Definitions for AI-Based Decision Support in Fluid Therapy: Call for Consensus

**DOI:** 10.2196/103138

**Published:** 2026-11-09

**Authors:** Audrius Andrijauskas, Tomas Jovaisa, Christer Svensen, Povilas Andrijauskas, Axel Kerroum, Vaidotas Marozas, Darius Dilijonas

**Affiliations:** 1 Clinic of Anaesthesiology and Intensive Care, Institute of Clinical Medicine, Faculty of Medicine, Vilnius University, Vilnius, Lithuania; 2 Department of Clinical Science and Education, Section of Anesthesiology and Intensive Care, Karolinska Institutet Södersjukhuset, Stokholm, Sweden; 3 Department of Anaesthesiology and Intensive Care, Vilnius University Hospital Santros Klinikos, Vilnius, Lithuania; 4 Department of Anesthesiology and Intensive Care, Lausanne University Hospital (CHUV), Rue du Bugnon, 46, Lausanne, Vaud, 1005, Switzerland, 41 213141111; 5 Biomedical Engineering Institute, Kaunas University of Technology, Kaunas, Lithuania; 6 Electronics Engineering Department, Faculty of Electrical and Electronics Engineering, Kaunas University of Technology, Kaunas, Lithuania; 7 Institute of Social Sciences and Applied Informatics, Kaunas Faculty, Vilnius University, Kaunas, Lithuania

**Keywords:** fluid therapy, hemodynamic monitoring, AI, clinical decision support systems

## Abstract

Intravenous fluid therapy is a cornerstone of optimizing body hydration, circulation, and perfusion, yet its rational implementation is limited by inconsistent clinical terminology. As fluid therapy moves toward AI-empowered clinical decision support systems (AI-CDSS), there is a critical need for high-quality, verified training data. The reliability of these systems is currently threatened by misleading data resulting from ambiguous definitions and the interchangeable use of nonsynonymous terms, such as “dehydration” and “hypovolemia.” This multidisciplinary panel-of-experts opinion article intends to start a discussion by identifying the key drawbacks in current terminology and proposes solutions, including a shift toward adequacy-based phenotypes and categorizing blood and water volumes as “insufficient,” “optimal,” or “excessive” rather than relying on context-sensitive quantitative measures such as “low,” “normal,” or “high.” Illustrative scenarios are used to show how inconsistent terminology and static quantitative labels could mislead an AI-CDSS. The proposed phenotype matrix is designed to provide a structured, systematic description of fluid requirements for specific clinical objectives that can be integrated into clinical algorithms and machine learning databases. An example of a fluid requirement assessment algorithm is used for exploring its applicability in AI training. The proposed terminology and phenotype matrix are provisional: they represent the opinion of a multidisciplinary expert panel and have not undergone formal consensus, clinical validation, or testing within an AI system. Broad consensus-building initiatives will follow.

## Introduction

### Background

Intravenous infusion of fluids has been a cornerstone of in-hospital treatment since the 19th century [1]. Although the use of fluid infusion is one of the most common interventions in medicine, its prescription remains one of the most difficult challenges in critically ill patients and in patients undergoing major surgery [2,3].

With the advent of minimally invasive and noninvasive monitoring techniques, alongside automated infusion systems, fluid therapy guided by clinical algorithms has become one of the major determinants of patient outcomes [4]. Timely and accurate assessment of whether a patient requires fluid administration, restriction, or removal is therefore crucial for optimizing both hydration and circulation [2].

However, this assessment is complicated by the complex and context-sensitive pathophysiology of fluid distribution and elimination. For instance, the relationship between extravascular and intravascular fluid volumes, effective circulating blood volume (stressed volume), and total blood volume is inconsistent [5]. Consequently, a patient may be hypotensive because of insufficient stressed volume, and fluid administration is therefore relatively indicated, despite total blood volume and total body water (TBW) being normal or even elevated [6].

Emerging evidence, algorithmic variability, and often contradictory research findings make rational fluid therapy challenging to implement [7]. Clinical decision support systems (CDSSs) based on practice guidelines and validated algorithms can optimize care [8], and real-time recommendations are increasingly delivered by AI-empowered clinical decision support systems (AI-CDSS) [9]. These tools derive evidence-based insights from patient data, medical literature, clinical guidelines, and real-time health analytics [9,10] and, particularly when integrated with automated infusion systems, may improve the efficacy and safety of fluid therapy [11-13]. Their recommendations will rely on rigorous analysis of up-to-date evidence within databases designed for machine learning [1].

However, AI applications can produce errors that can negatively impact patient care [14,15], and their reliability depends critically on the quality of data used to train them. In its 2021 report, *Ethics & Governance of Artificial Intelligence for Health* [16], and again during the generative AI boom in 2023‐2024 [17,18], the World Health Organization cautioned that precipitous adoption of untested systems could lead to severe medical errors, exacerbate bias, and exploit patient data.

Semantic interoperability requires the unambiguous collection, sharing, and use of data facilitated by adherence to terminology standards [19]. Inconsistent terminology in fluid therapy can introduce semantic errors when encoded into AI-CDSS, primarily because legacy medical records, clinical guidelines, and human clinicians frequently conflate distinct physiological concepts under generalized labels [3,6]. We argue that reliable deployment of AI-CDSS in fluid therapy requires a systematic revision of the terminology used in AI training databases.

To mitigate these risks, a new paradigm is required—one that combines clinically relevant and reliable definitions that satisfy the requirements of big data analytics. Such a dual approach is rarely used in clinical guidelines. In our multidisciplinary panel-of-experts opinion article, we therefore provide a brief introduction to the AI-CDSS and discuss the key drawbacks in current terminology and phenotype definitions in fluid therapy.

This article is addressed to 3 groups: clinicians who prescribe intravenous fluids in anesthesiology, intensive care, and emergency medicine; clinical informaticians and data scientists who construct the datasets and decision rules on which AI-CDSS depend; and developers of terminology standards, together with researchers designing fluid therapy trials, whose definitions determine whether evidence can be synthesized across studies.

Several revised terms and definitions are proposed with the aim of initiating discussion in the expert community, which may ultimately lead to consensus on their use in research, clinical practice, and machine learning databases.

### Development of the Proposals

The proposals presented here were developed as follows. The panel comprised 7 authors from 5 institutions in 3 countries: 5 clinicians in anesthesiology and intensive care, 1 researcher in applied informatics and data science, and 1 in biomedical engineering and electronics. The literature underpinning the proposals was identified by a narrative, nonsystematic search of PubMed covering the period from 1959 to 2026, using terms relating to fluid therapy, volume status and volemia, hydration status, hypovolemia and fluid overload, terminology and definitions, clinical decision support, AI, and machine learning; the reference lists of retrieved articles were screened for further sources. The definitions were generated by iterative drafting over 7 rounds, and disagreements were resolved by discussion during that process; no voting or other formal consensus procedure was used, and no external panel was consulted. Each proposed term was required to satisfy four criteria: that it be assessable using conventional, clinically practical methods rather than inferred from constructs that cannot be reliably measured at the bedside; that it refer unambiguously to a single compartment or function so that nonsynonymous terms cannot be used interchangeably; that it express functional adequacy rather than an absolute quantity; and that it be encodable as a discrete or ordinal label suitable for clinical algorithms and machine learning databases. The limitations of this approach are that the authors were not selected systematically, that agreement between them was never quantified, and that the composition of the panel may have influenced which distinctions were judged important. A formal Delphi process is proposed as the next step.

## Implementation of AI-CDSS in Fluid Therapy

The AI-CDSS combines structured medical knowledge with real-time patient information to generate individualized recommendations [9]. Most resemble modern expert systems—they embed knowledge, apply inference mechanisms, and generate recommendations within defined clinical contexts [20]. The most successful applications have emerged in constrained domains that rely on a single data source, such as radiology scans or laboratory values.

Fluid therapy, especially in critical care, is fundamentally different [12]: it is not a single-modality problem but a dynamic, context-sensitive “physiological negotiation,” in which decisions depend on a myriad of clinical data, including vital signs, hemodynamic parameters, laboratory findings, and their trends; screening results; comorbidities; ongoing treatments; timing; and clinical intent [3].

Automating such workflows requires hybrid systems that combine rule-based logic with data-driven pattern recognition, supervised by clinicians who validate and contextualize recommendations in real time [21].

Architecturally, AI-CDSS comprise a knowledge base with rules and an inference mechanism [22], which may use rule-based logic, Bayesian networks, decision trees, neural networks, natural language processing, case-based reasoning, or supervised and unsupervised learning, often in combination. They follow 2 broad paradigms: knowledge-based (classical expert) systems and data-driven (machine learning) systems [23-25].

Regardless of method, AI systems are likely to encode and propagate inconsistent terminology, phenotype definitions, and treatment concepts; model sophistication cannot compensate for conceptual ambiguity [26].

Terminology enters each architecture at a different point. In rule-based systems, an ambiguous term in the antecedent of a rule routes the patient down the wrong branch deterministically, although the error remains traceable. In supervised machine learning, inconsistent phenotype labels act as label noise that degrades performance silently and cannot be corrected by adding data from the same sources. Large language models (LLMs) absorb terminology from unstructured corpora in which nonsynonymous terms are already used interchangeably and lack the implicit clinical context needed to disambiguate them; mitigation therefore relies on ontology alignment, terminology normalization, and retrieval grounded in curated sources rather than relabeling. In closed-loop and automated infusion systems, the term defines the control target; an ambiguous target acts on the patient without intervening human judgment. Terminological precision is therefore a safety requirement, most critically where the technology is most autonomous.

Moreover, because different underlying states can appear similar on standard intensive care monitors, models such as random forest, XG Boost, or reinforcement learning–based sepsis “AI Clinicians” may misattribute physiological inputs [21].

## Terminology-Driven AI-CDSS Errors

### Overview

Fluid therapy is famously plagued by semantic overlapping—terms like fluid bolus, fluid challenge, fluid maintenance, and fluid replacement are frequently used interchangeably in practice despite representing entirely different clinical intents, rates, and volumes [27,28].

Terminology flaws that could cause errors in AI-CDSS recommendations include (1) mapping numerical measurements to semantic labels “low,” “normal,” or “high,” (2) conflating distinct physiological concepts under generalized labels and using nonsynonymous terms interchangeably; and (3) vague phenotype definitions and conceptual flaws in diagnostic algorithms.

### Mapping Numerical Measurements to Static Semantic Labels

Mapping numerical measurements to static semantic labels “low,” “normal,” or “high” bridges the gap between raw quantitative data and decision-making. Clinically most relevant body fluid compartments—intravascular (total blood volume that consists of unstressed and stressed blood volume) and extravascular (consists of extracellular and intracellular)—cannot be directly measured using clinically practical methods [29]. Mathematical models are not reliable. Thus, since clinical context-sensitive and case-specific “normal volume” cannot be determined, using the terms “low,” “normal,” or “high” implies a precise quantitative scale that does not exist in clinical practice.

What constitutes a safe (“optimal” or “sufficient”) vs dangerous (“insufficient” or “excessive”) volume is completely dynamic and context-sensitive [30]. A major challenge is that clinically practical methods to directly measure TBW, total blood volume, stressed, and unstressed blood volumes do not exist. However, even if such measurements were available, simple quantitative labels “low,” “normal,” or “high” would remain insufficient. Functional adequacy does not always correspond to “normal” absolute volume.

Simulated clinical scenarios illustrating how AI-CDSS could plausibly misinterpret static semantic labels “low,” “normal,” or “high” are presented in Multimedia Appendix 1. Thus, we propose the terminology shift from quantity-based static semantic labels “low,” “normal,” or “high” to adequacy-based terms “insufficient,” “optimal,” and “excessive.” Figure 1 illustrates its feasibility in a presumably AI-CDSS–compatible fluid requirement assessment algorithm. These are illustrative cases rather than empirical evidence: we are aware of no published data attributing errors in deployed AI-CDSS to terminological inconsistency in fluid therapy, and the mechanism described here should be read as a proposed risk rather than a documented one. We have not tested whether the proposed terms improve interobserver reliability or machine interpretability relative to the quantitative labels they replace. Establishing this is a necessary step and is set out in the conclusion.

**Figure 1.**
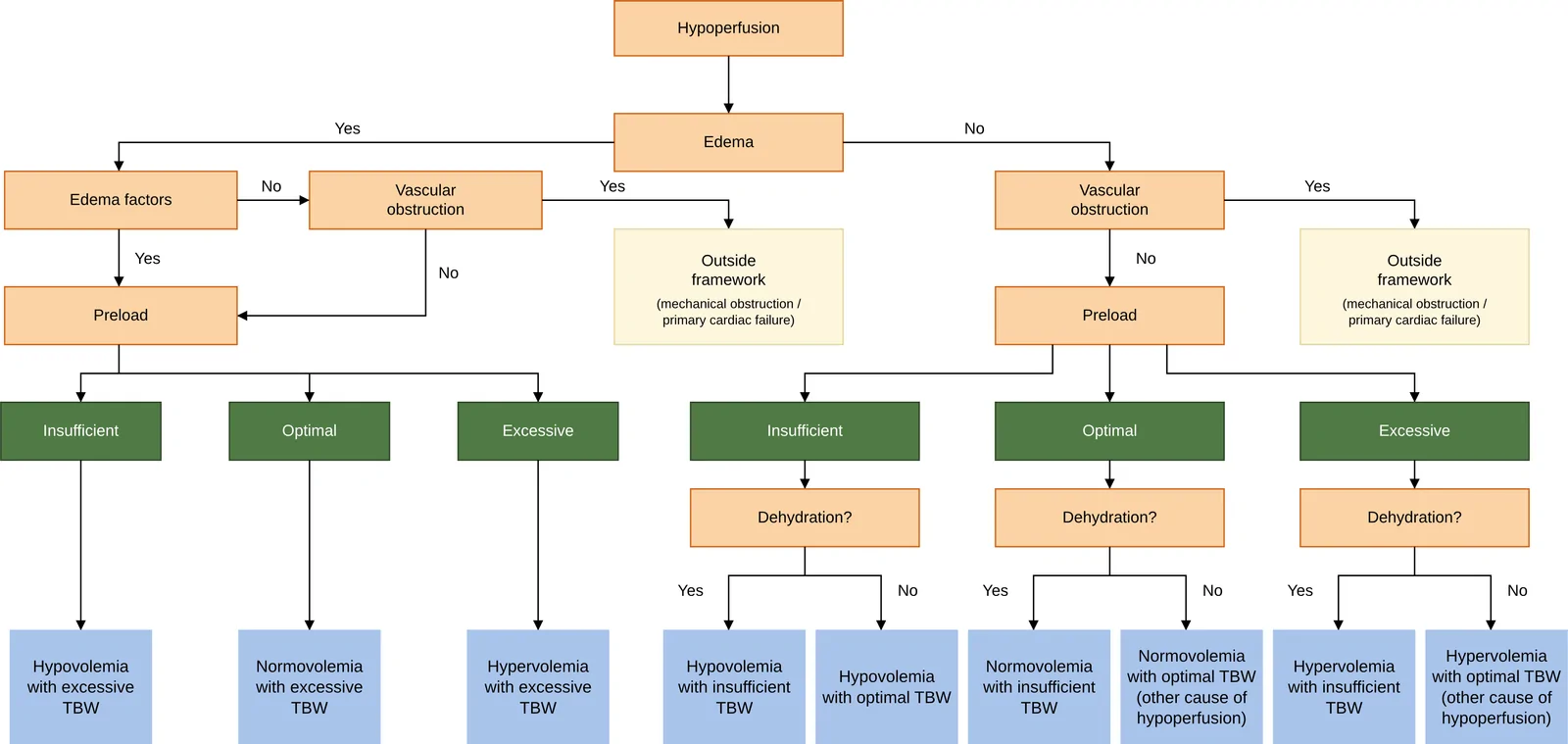
A fluid-requirement assessment algorithm defining perfusion of vital organs as the key diagnostic criterion in rational fluid management, proposing distinct phenotypes and exploring applicability in AI training. Qualitative phenotyping does not evaluate the degree of deviation within each category. “Edema factors”=factors predisposing to edema (eg, hypoalbuminemia). Light orange boxes indicate established physiological constructs and assessment steps; dark green boxes indicate the proposed terminology (“insufficient,” “optimal,” and “excessive,” replacing “low,” “normal,” and “high”), which is pending consensus and can potentially be operationalized using existing bedside measures (eg, stroke volume variation and inferior vena cava collapsibility for stressed volume; cumulative fluid balance, bioimpedance, and lung ultrasound B-lines for total body water [TBW]); these measurable tools are candidate input variables requiring validation for phenotype assignment; blue boxes indicate 9 resulting phenotypes; and yellow boxes indicate conditions outside the scope of this framework.

### Conflating Distinct Physiological Concepts Under Generalized Labels

The terms “euhydration,” “dehydration,” and “overhydration” are often interchangeably used (Table S1 in Multimedia Appendix 2) to express not one but several determinants of body fluid status—adequacy of TBW (body hydration), adequacy of intravascular volume (either total blood volume or stressed blood volume), and adequacy of intravascular fluid volume (plasma hydration). For example, if the term “dehydration” (insufficient TBW) is used in databases used for training AI to indicate “hypovolemia,” or vice versa, it may lead to errors in AI-CDSS–generated clinical recommendations. Similarly, errors occur when the term “hypervolemia” is used to indicate “overhydration” (excessive TBW), or vice versa. Table 1 demonstrates the comparative framework of the linguistic impact on the safety of AI-CDSS recommendations using the example of frequently used terminology in fluid therapy.

These limitations have been recognized in fluid-balance research itself, where inconsistent operational definitions have been identified as a barrier to synthesizing evidence across studies [31], as well as in broader analyses in both machine learning and language recognition research [32] and in broader analyses of clinical big data [33]. For AI-CDSS applications in fluid management, such ambiguity is not merely semantic; it has the potential to affect model training inference and safety.

**Table 1.** Comparative framework of the linguistic impact on AI safety metrics.

Terminology flaw/ambiguity	AI misinterpretation	Expected clinical end point	AI-recommended end point (error)	Patient safety risk
“Fluid bolus” used instead of “fluid challenge”	The system assumes high-volume macro-hemodynamic correction	Small volume (100‐200 mL) to test responsiveness	Large volume (500‐1000 mL) bolus	Hydrostatic pulmonary edema/fluid overload
“Fluid overload” is used interchangeably with “hypervolemia”	The system treats intravascular volume excess as total-body tissue third spacing	Continuous monitoring or restricted maintenance	Aggressive loop diuretics or ultrafiltration	Iatrogenic hypotension and acute kidney injury
“Maintenance” vs “replacement”	The system fails to differentiate baseline physiological loss from active pathological output (gastrointestinal bleed/fistula)	Standard crystalloid tailored to baseline caloric/homeostatic needs	Hypotonic maintenance fluid administered at high rates	Severe hyponatremia and cerebral edema

### Vague Definitions of Phenotypes and Conceptual Flaws in Diagnostic Algorithms

The clinical algorithms, including diagnostic road mapping, are commonly used in fluid management and therefore are important for training AI. The primary goal of fluid therapy is optimization of perfusion of vital organs by improving macrocirculatory and microcirculatory flow, largely through modulation of intravascular and extravascular fluid volumes, that is, by optimizing the effective circulating blood volume (volume status or “volemia”) by changing the plasmadilution and TBW (hydration status) by transcapillary fluid equilibration [30]. This requires clear, context-sensitive definitions linking perfusion, effective circulating blood volume, total blood volume, and TBW, as well as a coherent description of the relationship between intravascular and extravascular fluid compartments.

However, many commonly used definitions lack physiological precision and are only loosely connected to perfusion (Table 2). Such conceptual gaps may be manageable in narrative reasoning but become critical when formalized in algorithms. Reliable AI-CDSS–compatible diagnostic algorithms are of key importance for prescribing rational fluid therapy. We propose an algorithm where—uniquely—the conventional clinical signs of hypoperfusion serve as a trigger for an algorithmic pathway to determine its causes (Figure 1). The derived diagnosis in terms of fluid requirement—the need for fluid or fluid removal—provides insights into rational fluid management. All terms and definitions within an algorithm are related to parameters that are measurable or assessable using clinically practical methods. We also suggest the use of the revised definitions and fluid status phenotypes (*see the next section*) to improve the reliability of the algorithm. The feasibility of the algorithm will be evaluated in our future research.

**Table 2.** Definitions pertaining to volemia.

Source	Definitions
De Backer et al, 2022 [30]	“A functional definition of normovolemia may be the filling of the heart that ensures cardiac output and oxygen delivery. From that perspective, supine humans are normovolemic, in that a maximal venous oxygen saturation (SvO_2_) is established.”
Secher et al, 2005 [34]	“Hypovolemia could be defined as a decrease in blood volume resulting from sufficient loss of blood, plasma, and/or plasma and water to trigger the sympathetic compensatory response to maintain hemodynamic stability.” “When those mechanisms start to fail, hypovolemia may be characterized by a reduced cardiac preload.”
Vincent et al, 2018 [6]	“Hypovolemia refers to a low intravascular blood volume, wherein mean systemic filling pressure is low and there is a net flux of water and electrolytes out of the interstitial and tissue spaces into the blood stream.”
Vincent et al, 2018 [6]	“Hypervolemia refers to excessive blood volume, wherein mean systemic circulatory pressure is high, causing edema.”

Figure 1 sets this algorithm out in full. Its entry condition is hypoperfusion; the pathway then tests for edema and for factors predisposing to it, for vascular obstruction, and for preload and terminates in the phenotypes described in the next section. The figure is color-coded to separate what is established from what we propose: light orange marks established physiological constructs and the assessment steps built on them, dark green marks the proposed adequacy-based terminology that replaces the quantitative labels and that remains consensus-pending, blue marks the resulting phenotypes, and yellow marks the two branches that fall outside this framework—mechanical obstruction and primary cardiac failure.

The alarming concern is that—in databases—the terms “effective circulating volume” and “stressed volume” are often used interchangeably. While physiologically related, the former originates from classical physiology and the latter from compartmental modeling [6]. Alternating between such terms within a single article, or across datasets, complicates both human interpretation and machine parsing. When AI systems or LLMs are trained on clinical data or guidelines that use nonsynonymous terms interchangeably, they lack the “implicit clinical context” to decouple them, leading to specific, dangerous recommendation errors.

More concerning is the routine interchangeable use of the terms “fluid overload” and “hypervolemia.” In 2018, experts recommended abandoning the term “fluid overload” because of its imprecision and frequent confusion with hypervolemia, warning that such ambiguity may contribute to inappropriate fluid management [6]. Despite this, the terms continue to be used interchangeably in both clinical practice and the literature [30,35]. Notably, even authors who proposed to avoid the term “fluid overload” in a paper published in 2018 [6] subsequently coauthored a 2022 paper in which the same term was used to describe an adverse effect of fluid infusion [30].

Another example concerns the term “blood volume,” which is often used interchangeably with “stressed volume” or “effective circulating volume,” despite referring to different physiological concepts. In some integrative interpretations of volume status and extravascular lung water, “blood volume” is effectively used to denote stressed volume [30]. However, total blood volume comprises both unstressed and stressed (effective circulating) components. If an AI-CDSS algorithm interprets “low blood volume” as synonymous with “low effective circulating volume” [6], it may recommend fluid administration even when total blood volume is normal and the underlying issue is altered vascular tone rather than insufficient intravascular volume. Such semantic imprecision can therefore translate directly into inappropriate therapeutic suggestions.

Further complexity arises with the term “functional” or “relative hypovolemia,” which describes circulatory states characterized by insufficient stressed volume that leads to insufficient cardiac output despite a normal total blood volume, for example, when mean circulatory filling pressure decreases due to vasoplegia [6]. Here, the problem lies not in loss of total blood volume but in altered vascular tone and blood distribution. Without precise terminology, such distinctions are easily obscured.

The persistence of these ambiguities likely reflects both lack of consensus and entrenched clinical habit. A habit of using inappropriate terms in medicine comes from the time when their use was based on the limited knowledge of the processes they were referring to. Decades ago, clinicians labeled the NaCl 0.9% solution as “normal saline” and “physiological solution.” These labels are still frequently used, although they are fundamentally misleading because of the inherently abnormal and unphysiological features of this solution [36]. While such linguistic inertia may seem harmless, in the context of AI training, it embeds outdated or imprecise concepts directly into structured datasets and decision logic.

## Revisited Definitions and Phenotypes

### Overview

We propose the overarching term “fluid status” to describe body hydration status in terms of the functional adequacy of the estimated or measured TBW. The TBW is the sum of intravascular (plasma) and extravascular (tissue) fluid volumes. Prescribing fluid therapy requires information on whether fluid volumes in the intravascular and extravascular compartments meet functional requirements. Because the adequacy of fluid in these compartments correlates poorly, it must be assessed separately. This distinction necessitates clearly defined, combined phenotypes (Figure 2).

Figure 2 presents this distinction as a 2-axis matrix. The vertical axis represents volume status, from hypovolemia through normovolemia to hypervolemia, and the horizontal axis represents hydration status, from dehydration through euhydration to edema. The shaded green zone at the intersection denotes the region of combined adequacy, in which both axes are optimal; because both axes are continuous rather than categorical, its boundary is a gradient rather than a threshold. The red dots mark the fluid status of a single patient at successive time points during an observation period so that the sequence conveys the direction and the speed of travel across the matrix rather than a single state. Read this way, the figure separates a patient who lies outside the green zone but is moving toward it from one who is drifting away from it, a distinction that no single categorical label can carry.

**Figure 2.**
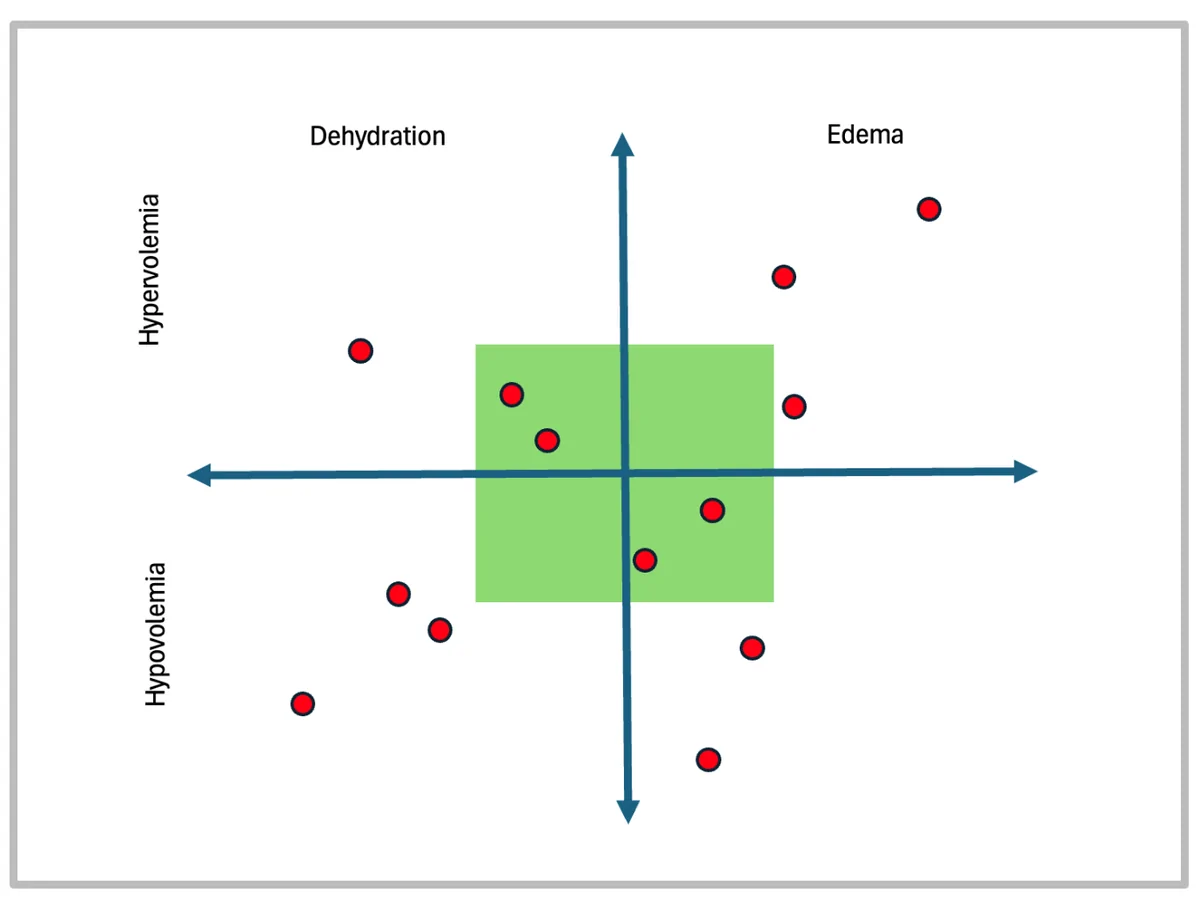
An illustrative fluid status encoding scheme. Red dots indicate the patient’s fluid status at different time points during the observation period. Status within a “green zone” is considered as optimized fluid status, especially in critically ill patients.

### Volume Status (Volemia)

#### Overview

We emphasize that perfusion is dependent on the adequacy of both volemia and TBW—the main determinant of tissue hydration status. For example, hypoperfusion can be present with any volume status if TBW is excessive because edema is mechanically obstructing microcirculation and increasing the distance from capillaries to cells; sufficient perfusion can be present with hypervolemia in conditions with plasma hyperosmolality.

We propose defining volemia as the clinical context-sensitive adequacy of total blood volume, stressed volume, and cardiac function (stroke volume and cardiac output) to ensure optimal vital organ perfusion, provided there are no edema or dehydration predisposing factors (Figure 1; Table 3). Mechanical obstruction and primary cardiac failure represent separate mechanisms of hypoperfusion and are outside this framework.

#### Normovolemia (Euvolemia)*:*

Optimal perfusion is maintained by an adequate combination of total blood volume, stressed volume, and cardiac function, provided there are no edema-predisposing factors such as hypoalbuminemia.

**Table 3.** Fluid status phenotype matrix.

Volume status (intravascular fluid compartment)	Hydration status (extravascular fluid compartment)		
	Dehydration (insufficient TBW^a^)	Euhydration (optimal TBW)	Edema (excessive TBW)
Hypovolemia	Hypovolemia with dehydration	Hypovolemia with euhydration	Hypovolemia with edema
Normovolemia	Normovolemia with dehydration	Neutral (optimal) state	Normovolemia with edema
Hypervolemia	Hypervolemia with dehydration	Hypervolemia with euhydration	Hypervolemia with edema

a TBW: total body water.

#### Hypovolemia

Insufficient perfusion (hypoperfusion) is due to an inadequate combination of total blood volume, stressed volume, and cardiac function, provided there are no edema predisposing factors.

#### Hypervolemia

Impaired perfusion is due to an inadequate combination of total blood volume, stressed volume, and cardiac function that leads to venous congestion—the primary cause of edema—provided there are no dehydration-predisposing factors such as plasma hyperosmolality.

### Hydration Status

The term “hydration status” refers to the adequacy of extravascular fluid volume, mainly determined by the adequacy of TBW. Because plasma accounts for only 17% of extracellular volume, whereas interstitial fluid volume comprises approximately 50% to 60% [37,38], relatively small changes in interstitial fluid can substantially alter TBW.

In contrast, even considerable shifts in intravascular volume—total blood volume—may have little impact on TBW. Accordingly, TBW may remain unchanged when fluid excess in one compartment offsets a deficit in another. This can occur, for example, when a reduction in total blood volume is accompanied by a corresponding increase in extravascular volume due to capillary leak. Consistent with this, studies have shown that total blood volume may remain normal after preoperative fasting [39] despite obvious reduction in TBW. Our proposed definitions of hydration status are as follows (Figure 1; Table 3): *euhydration*, optimal TBW; *dehydration*, insufficient TBW; and *edema* (overhydration), excessive TBW. Since there is a weak relationship between extravascular fluid volume and effective circulating (“stressed”) blood volume [30], oliguria occurs early during dehydration but appears late in hypovolemia [5]. *Edema* may coexist with any state of volemia, and its presence does not exclude a need for fluids [6]. Likewise, an increase in TBW is not systematically accompanied by an increase in total and/or stressed blood volume [30]. Plasma volume represents a link between extravascular fluid volume and total blood volume. These volumes are normally in parallel but diverge in pathologic states—for example, edema in heart failure is present with reduced intravascular fluid volume [38]. Edema may coexist even with hypovolemia when there are factors that predispose to edema. We use the term “edema factors,” which also appears in Figure 1, for the factors that predispose to interstitial fluid accumulation independently of intravascular volume, for example, positive fluid balance, the period following the salvage or resuscitation phase of fluid therapy, capillary leak, hypoalbuminemia, hypo-osmolality, and kidney or liver disease.

These relationships are not reciprocal. Excessive stressed volume may or may not cause heart failure; excessive TBW always manifests as edema but may or may not cause heart failure; and heart failure may or may not cause edema. Heart failure and excessive stressed volume are frequently but not invariably associated: heart failure with preserved ejection fraction may occur with a reduced effective circulating volume, in which case the stressed volume is insufficient rather than excessive.

We further propose that the term “fluid balance” be qualified by specifying compartmental adequacy, for example, “positive fluid balance with hypervolemia and edema” or “negative fluid balance with euvolemia and edema.” This distinction is clinically relevant, as intravascular and extravascular compartments often require different therapeutic strategies.

Because an optimal TBW (optimal hydration state), optimal total blood volume, and optimal stressed blood volume are individualized rather than absolute, adequacy is defined here functionally and anchored to the adequacy of vital organ perfusion, which has established clinically practical measurable correlates—perfusion indices—urine output, serum lactate and lactate clearance, and capillary refill time. Position on each axis of the phenotype matrix can then be estimated from candidate input variables that are already acquired at the bedside and that would themselves require validation for phenotype assignment, as set out below. The formal interrater and intersystem reliability of the resulting composite phenotype has not been tested, and establishing it is a necessary step before these labels are used to train or evaluate an AI-CDSS.

In clinical practice, especially in critical care, a dynamic assessment reflecting the trajectory of a patient’s condition is essential. Integrating definitions of volemia and hydration into a phenotype matrix that provides a structured and systematic description of fluid status and related fluid requirements (Table 3) may therefore be valuable for practice, research, and AI-CDSS applications.

An illustrative encoding scheme reflects a static snapshot of fluid status (Figure 2). For structured data encoding, the matrix could be operationalized in 2 ways. A categorical approach assigns each axis a discrete class (eg, hypovolemia, normovolemia, or hypervolemia; dehydration, euhydration, or edema), yielding the 9 combinatorial phenotypes shown in Table 3. Alternatively, an illustrative graded ordinal encoding scheme scores each axis on a quantitative scale (eg, 1‐10, with 5 representing the optimal midpoint), producing a two-digit phenotype code such as “V3H8” (V for volemia and H for hydration status) that more closely reflects the continuous distribution of fluid status illustrated in Figure 2, rather than forcing patients into discrete bins. This ordinal scheme is illustrative: its anchors and measurement properties remain to be established. Ordinal positions could be anchored to variables measured at the bedside—dynamic indices of fluid responsiveness such as stroke volume variation or inferior vena cava collapsibility for the stressed volume axis and cumulative fluid balance, bioelectrical impedance, or lung ultrasound B-line count for the extravascular water (TBW) axis—providing a practical bridge between existing bedside tools and machine-readable phenotype coding. An example of an AI-CDSS–compatible fluid requirement assessment algorithm defining distinct phenotypes and illustrating its applicability in training AI is presented in Multimedia Appendix 3. The variables on each axis are acquired by clinically practical methods, so this provides a potential basis for integration. We have not implemented or tested such integrations but propose it as future research.

## The Future of AI-CDSS

Terminological variability [6,27] is a recognized challenge in AI training and language processing [32,33]. In fluid management, this problem is particularly consequential: ambiguous, inconsistent, or physiologically imprecise terms risk being encoded directly into datasets and algorithms. Achieving uniform terminology therefore requires not only linguistic clarification but also genuine clinical consensus. Importantly, definitions must be practical, grounded in parameters that can be reliably assessed in routine care rather than inferred from constructs that cannot be reliably measured in clinical practice [30]. Thus, we propose replacing the terms “low,” “normal,” and “high” that imply a precise quantitative scale that does not exist in clinical practice with definitions of adequacy—“insufficient,” “optimal,” and “excessive” (Table 4).

AI integration into CDSS has clear potential to improve diagnostic accuracy, prognostic assessment, and patient safety [40,41]. However, machine learning models, including LLMs, derive statistical associations from existing data and cannot resolve conceptual inconsistencies or physiological ambiguity embedded within it—a nontrivial limitation in heterogeneous environments such as critical care [42,43].

Anesthesiology and critical care, being protocolized and data-intensive, are well suited for AI support [44], yet AI systems cannot replace clinician judgment in complex or atypical scenarios [45,46]. Model opacity remains a major barrier to adoption; explainable AI (XAI) aims to make model reasoning understandable to clinicians, but trustworthy XAI–CDSS require a user-centered approach that addresses clinician needs, workflow integration, and usability [9].

Rapidly developing LLM-based applications offer an opportunity to leverage large-scale medical knowledge [47-49], but their effectiveness depends on semantic consistency: without harmonized terminology and precise phenotype definitions, AI systems may amplify rather than reduce variability.

Before AI can reliably guide fluid therapy, expert consensus on terminology and phenotype definitions is essential, followed by robust, evidence-based clinical algorithms suitable for structured implementation [11]. Clear definitions are prerequisites for trustworthy datasets, reliable models, and safe clinical decision support.

Practical barriers also remain. The real-time curation of multidimensional data streams into clinical decision support—the efferent limb of AI-CDSS—is often ignored during initial validation, and legal and commercial barriers to clinical databases limit studies of fairness and generalizability [12].

**Table 4.** Illustrative comparison of potential AI interpretations using quantitative versus adequacy-based labels.

AI parameter labeling	AI interpretation of data	AI-recommended fluid management	Clinical outcome/error
“Low” blood volume^a^	*Absolute*: Total blood volume is lower than a healthy person’s “normal” value	Continue crystalloid fluid boluses (rescue phase of fluid therapy)	Worsened perfusion: cardiac output stays low, fluid accumulates in tissues (capillary leak), and compresses microcirculation
“Insufficient” blood volume^a^	*Functional*: Total blood volume is higher than normal value, but functionally “insufficient” for maintaining “necessary” stressed blood volume	Stop boluses, start maintenance (optimization), start low dose vasopressors (eg, norepinephrine)	Optimized perfusion: cardiac output increases, microcirculation improves, fluid accumulation in tissues slows down
“Low” stressed blood volume^b^	*Absolute*: Metrics (TEE^c^) are lower than a healthy person’s normal value (inferior vena cava measurement)	Dehydration-related hypovolemia: Give fluid bolus	Worsened perfusion: stroke volume and cardiac output drop further, fluid accumulates further (chronic heart failure), and edema compresses microcirculation
“Excessive” stressed blood volume^b^	*Functional*: Metrics (TEE) are lower than “normal,” but individually “excessive” for failing heart to cope.	Overhydration (edema): Hold fluids/cautiously give diuretics	Optimized perfusion: cardiac output increases, microcirculation improves, fluid accumulation in tissues resides

a Context: hypoperfusion and arterial hypotension in septic shock after 3 L of crystalloid.

b Hypoperfusion (markers), stroke volume, and cardiac output (TEE) below “normal” without hypotension routinely measured after 4 h of ongoing minimally bleeding plastic surgery in an 88-year-old man with peripheral nerve block and light sedation.

c TEE: transesophageal echoscopy.

## Conclusions

Since the AI-supported decision-making era in fluid therapy has already begun, there is an urgent need to revise and to define, by expert consensus, the relevant terminology and definitions of phenotypes for use in databases that train AI-CDSS. This paper aims to initiate a debate. Further consensus-building efforts are required to validate the proposed solutions to a complex problem.

We propose a staged pathway for that work: first, expert consensus and ontology development through a formal Delphi process; second, operational definition of each term and testing of interrater reliability; third, retrospective validation against clinical datasets; fourth, computational validation within an AI-CDSS; and finally, prospective evaluation of AI-CDSS and, ultimately, of automated therapy.

The present paper provides the preliminary expert-derived framework from which the first stage—formal consensus and ontology development—can proceed. Semantic reproducibility and clinical validity must be established separately at each subsequent stage because a phenotype can be perfectly reproducible and still encode an oversimplified physiological model.

## Supplementary material

10.2196/103138 Multimedia Appendix 1 Simulated clinical scenarios illustrating how AI-empowered clinical decision support systems could plausibly misinterpret the static semantic labels “low,” “normal,” and “high,” with the possible clinical errors and outcomes.

10.2196/103138 Multimedia Appendix 2 Examples of interchangeably used terms that are not synonyms in fluid therapy.

10.2196/103138 Multimedia Appendix 3 Fluid status assessment algorithm: background concepts, definitions, and a simulated stepwise application to a capillary leak scenario within an envisioned AI-empowered clinical decision support system.

## Abbreviations

AI-CDSS: AI-empowered clinical decision support system
CDSS: clinical decision support system
LLM: large language model
TBW: total body water
XAI: explainable AI
