# Supplementary material for: Revising Terminology and Phenotype Definitions for AI-Based Decision Support in Fluid Therapy: Call for Consensus
Source: J Med Internet Res. 2026 Nov 9;28:e103138. doi: 10.2196/103138 (PMC13747319; doi:10.2196/103138)
Supplement: Supplementary file 1 [file jmir-v28-e103138-s001.docx]

# **Multimedia Appendix 1**

Simulated clinical scenarios illustrating how artificial intelligence–empowered clinical decision support systems (AI-CDSS) could plausibly misinterpret static semantic labels “low,” “normal,” and “high” (Table 4 in the main text). Illustrative scenarios are presented alongside possible clinical errors.

## Illustrative scenario 1

### Clinical scenario

The patient with sepsis-related vasoplegia is treated for severe distributive shock in the emergency department. The compliance of the venous system is high, the intravascular space is significantly increased that results in increase of unstressed blood volume, and decrease in stressed volume [1]. There is a need for more blood in the unstressed volume to create enough stressed volume to maintain the driving pressure that would be sufficient for maintaining necessary cardiac output for optimal vital organ perfusion. In compliance with practice guidelines for septic shock, in the rescue phase of fluid therapy the patient has received about 3 L of intravenous crystalloid solution but remains in shock condition. The care provider turns to the AI-CDSS for advice regarding further fluid prescription.

How static semantic label “normal” and “high” mislead an AI-CDSS?

Given the 3 L of fluid infused as bolus in the rescue phase, the AI-estimated total blood volume is high or at least normal compared to a healthy resting human estimate, and thus the AI-CDSS advice is—"Stressed volume is “Normal” or “High”. Stop fluids. Initiate high-dose vasopressors.”

The possible clinical error because of AI-CDSS fail while the total blood volume might be normal—not high since fluids are leaving circulation due to capillary leak—it is functionally insufficient and thus cannot maintain sufficient stressed volume for sufficient cardiac output. Stopping fluids prematurely and starting high dose vasopressor infusion causes severe organ hypoperfusion [2].

The possible outcome if AI-CDSS’s advice is followed—The patient’s condition deteriorates further with systemic organ hypoperfusion leading to multiple organ failure.

## Illustrative scenario 2

### Clinical scenario

The patient with acute respiratory distress syndrome (ARDS) and diastolic dysfunction (“stiff heart”) is treated in the intensive care unit. The ventricles and capacitance vessels are noncompliant. Because the circulation system cannot stretch, even a small amount of infused fluid generates dangerously high systemic filling pressures [3].

How static semantic label “low” mislead an AI-CDSS?

The AI-estimated absolute total blood volume, and stressed volume are low compared to standard population metrics due to restrictive fluid therapy in ARDS.

The AI-CDSS advice is “Stressed volume is low, and thus fluid bolus is recommended”.

The possible clinical error because of AI-CDSS fail: Because the AI assumes that “low” means “needs more”, it does not acknowledge that this volume is functionally excessive for the stiff cardiovascular system.

The possible outcome if AI-CDSS’s advice is followed—The patient’s condition deteriorates because the recommended fluid bolus yields either no increase or even decrease in stroke volume (the flat part of the Frank-Starling curve), and instead causes an immediate backward pressure spike, forcing fluid into the lungs and worsening ARDS [4].

## **References**

1. Spiegel R. Stressed vs. unstressed volume and its relevance to critical care practitioners. Clin Exp Emerg Med. 2016 Mar;3(1):52-4. PMID: 27752616. doi: 10.15441/ceem.16.128.

2. Fot EV, Khromacheva NO, Ushakov AA, Smetkin AA, Kuzkov VV, Kirov MY. Optimizing fluid management guided by volumetric parameters in patients with sepsis and ARDS. Int J Mol Sci. 2023 May 15;24(10):8768. PMID: 37240114. doi: 10.3390/ijms24108768.

3. Adamu UG, Muponda B, Tsabedze N. Optimal fluid management strategies in patients with heart failure: a systematic review and meta-analysis of randomized controlled trials. Front Cardiovasc Med. 2025 Oct 31;12:1636862. PMID: 41246014. doi: 10.3389/fcvm.2025.1636862.

4. Ziaka M, Exadaktylos A. Fluid management strategies in critically ill patients with ARDS: a narrative review. Eur J Med Res. 2025 May 20;30(1):401. PMID: 40394685. doi: 10.1186/s40001-025-02661-w.
