# Supplementary material for: Revising Terminology and Phenotype Definitions for AI-Based Decision Support in Fluid Therapy: Call for Consensus
Source: J Med Internet Res. 2026 Nov 9;28:e103138. doi: 10.2196/103138 (PMC13747319; doi:10.2196/103138)
Supplement: Supplementary file 2 [file jmir-v28-e103138-s002.docx]

**Multimedia Appendix 3**

**Table S1.** Examples of interchangeably used terms that are not synonyms in fluid therapy.

Fluid bolus, fluid challenge, fluid replacement, maintenance infusion;

Edema, fluid accumulation, positive fluid balance;

Fluid status, hydration status, volume status, volemia;

Blood volume, intravascular volume, circulating volume, effective circulating volume;

Hypovolemia, dehydration, low intravascular volume, low circulating volume, low effective circulating volume, low blood volume, low plasma volume, low circulating fluid volume, low intravascular volume, as well as true, real, relative and functional hypovolemia;

Hypervolemia, fluid overload, high effective circulating volume, high intravascular volume, high circulating volume, high circulating fluid volume, high blood volume, high plasma volume, as well as true, real, relative and functional hypervolemia.
