# Supplementary material for: Revising Terminology and Phenotype Definitions for AI-Based Decision Support in Fluid Therapy: Call for Consensus
Source: J Med Internet Res. 2026 Nov 9;28:e103138. doi: 10.2196/103138 (PMC13747319; doi:10.2196/103138)
Supplement: Supplementary file 3 [file jmir-v28-e103138-s003.docx]

# **Multimedia Appendix 2**

## Fluid status assessment algorithm

The main objective of developing a new fluid requirement assessment algorithm using the revised terminology was to explore theoretically its clinical feasibility and applicability in clinical decision support systems (CDSS) with AI applications (AI-CDSS) for fluid therapy. Since its implication requires extended monitoring, it is applicable only to critically ill patients and those undergoing major surgery. The algorithm applies to optimization, stabilization, and de-escalation phases, but does not apply to the rescue phase of fluid therapy [1,2].

The background concepts, definitions, and terms of the proposed algorithms:

The proposed algorithms provide decision-making pathways for assessing fluid requirements and answering the fundamental question “is there a need for fluid or fluid removal?”. Fluid status is represented by total body water (TBW). However, prescribing correct fluid management requires answering the question “is there an insufficient fluid or excessive fluid volume in circulation or in tissues, or in both fluid compartments?”. Therefore, at the end of the algorithmic pathways there are conclusions relevant to the treatment, eg, in the pathway hypoperfusion–edema–insufficient preload the conclusion is hypovolemia with excessive TBW” which suggests that fluid restriction rather than removal is the safe option for reducing the excess of extravascular fluid because fluid removal may further deteriorate hypovolemia and related hypoperfusion.

Diagnostic and treatment recommendations are not within the scope of this paper.

Hypoperfusion of vital organs requires urgent treatment. Thus, in the proposed algorithm, perfusion status is the starting point in the diagnostic pathway (Figure 1 in the main text). Since hypoperfusion can be caused by hypo- and hypervolemia, as well as edema, the next steps are the assessment of hydration (TBW) and volemia (preload).

Edema is the main determinant of excessive TBW. Thus, if edema is present, TBW is excessive in association with any volume status, eg, when there are factors predisposing to edema, TBW is excessive even in hypovolemic states (Figure 1 in the main text). Conversely, if edema is not present, TBW is optimal in association with any volume status.

Factors predisposing to edema (excessive TBW) include positive fluid balance, conditions after the salvage/resuscitation phase of fluid therapy, capillary leak, hypoalbuminemia, hypo-osmolality, kidney, and liver diseases.

The theoretical exploration of clinical feasibility and applicability of the algorithms in AI-CDSS includes a simulated stepwise evaluation of fluid status using a simulated user interface.

## Capillary leak scenario pathway

Sepsis is characterized by a combination of a decrease in vascular tone, affecting both arterioles and venules, myocardial depression, alterations in regional blood flow distribution and microvascular perfusion and capillary leak due to increased vascular permeability [3]. Such conditions are associated with edema and hypoperfusion. The following clinical scenario represents the post-resuscitation phase of fluid therapy and critically ill patient recovering after septic shock.

The proposed steps of fluid requirement assessment which are presumed to be compatible with the user interface of an envisioned AI-CDSS platform:

Physician enters/downloads patient’s demographic data and history, as well as data from intensive care unit chart including fluid balance and vital signs and results from laboratory, physical and instrumental investigations (for example, dynamic indices such as pulse pressure/stroke volume variation or inferior vena cava collapsibility for stressed volume; cumulative fluid balance, bioimpedance, or lung ultrasound B-lines for TBW). The AI-CDSS performs an assessment of fluid requirements.

The AI analyzes data by following the algorithm (Figure 1 in the main text) that was used to train the AI.

The AI-CDSS software summarizes data analysis and highlights the presence of hypoperfusion which is the starting point of the algorithm (hypoperfusion pathway is depicted in Figure 1 in the main text).

Since edema per se can cause hypoperfusion, the next step is to check for signs of edema and/or factors predisposing to edema (excessive TBW).

The presence of edema and factors predisposing to edema (sepsis-related capillary leak and state after aggressive fluid loading in resuscitation phase) is sufficient for concluding that TBW is excessive, and measures for reducing extravascular fluid are indicated.

Since both hypo- and hypervolemia can be confounding factors that cause edema, and thus may complicate fluid management strategies, the next step is the assessment of volemia, eg, by evaluating data from preload measurements.

Signs of hypovolemia (insufficient stressed volume), most likely due to sepsis-related vasoplegia, are detected.

The AI analyzes and highlights the strength of evidence behind the algorithmically proposed diagnosis of “Hypovolemia with excessive TBW” and provides some advice “Cautious removal of excessive fluid rather than aggressive is the best strategy in terms of safety”.

Alternative algorithms might be preferred in some clinical scenarios.

The AI-CDSS advised clinical strategy is based on the review of up-to-date information in the dedicated database. Recommendations could include the following: (a) treat the main cause (sepsis), (b) maintain plasma osmolality (focus on albumin), (c) minimize detrimental effect of vasopressors on microcirculation such as using multiple drugs rather than one, (d) assess fluid responsiveness using dynamic parameters rather than static parameters, (e) the most specific and sensitive target parameters for volume and hydration status are recommended from the operator's pre-entered list of available parameters.

## **References**

1. Malbrain M, Van Regenmortel N, Saugel B, De Tavernier B, Van Gaal PJ, Joannes-Boyau O, et al. Principles of fluid management and stewardship in septic shock: it is time to consider the four D's and the four phases of fluid therapy. Ann Intensive Care. 2018 May 22;8(1):66. PMID: 29789983. doi: 10.1186/s13613-018-0402-x.

2. Vincent JL. Fluid management in the critically ill. Kidney Int. 2019 Jul;96(1):52-7. PMID: 30926137. doi: 10.1016/j.kint.2018.11.047.

3. Messina A, Bakker J, Chew M, De Backer D, Hamzaoui O, Hernandez G, et al. Pathophysiology of fluid administration in critically ill patients. Intensive Care Med Exp. 2022 Nov 4;10(1):46. PMID: 36329266. doi: 10.1186/s40635-022-00473-4.
